# Relationships between depersonalization-derealization symptoms and separation anxiety in adult patients with mood and anxiety disorders

**DOI:** 10.3389/fpsyt.2025.1565217

**Published:** 2025-05-08

**Authors:** Stefano Pini, Benedetta Nardi, Barbara Carpita, Giada Lorenzi, Marco Mula, Barbara Milrod, Gabriele Massimetti, Ivan M. Cremone, Chiara Bonelli, Katharina Domschke, Miriam Schiele, Liliana Dell’Osso, David S. Baldwin

**Affiliations:** ^1^ Department of Clinical and Experimental Medicine, University of Pisa, Pisa, Italy; ^2^ Institute of Medical and Biomedical Education, St George’s University Hospitals National Health Service (NHS) Foundation Trust and City St George’s University of London, London, United Kingdom; ^3^ Albert Einstein College of Medicine, PRIME (Psychiatric Research Institute of Montefiore Einstein), New York, NY, United States; ^4^ Department of Psychiatry and Psychotherapy, Medical Center – University of Freiburg, Faculty of Medicine, University of Freiburg, Freiburg, Germany; ^5^ Clinical and Experimental Sciences, University of Southampton Faculty of Medicine, Southampton, United Kingdom; ^6^ University Department of Psychiatry and Mental Health, University of Cape Town, Cape Town, South Africa

**Keywords:** separation anxiety, adult separation anxiety, depersonalization, derealization, suicidality, dissociative symptoms

## Abstract

**Aims:**

To establish the relationship between depersonalization/derealization symptoms (DPs), as assessed by different standardized DP scales, and separation anxiety in a sample of outpatients with anxiety and mood disorders as a primary diagnosis (n=156). We hypothesized that patients with high levels of separation anxiety had more frequent, severe, and clinically relevant DP symptoms than those with low levels of separation anxiety.

**Methods:**

A consecutive sample of 156 outpatients with mood and anxiety disorders was evaluated by the Structured Clinical Interview for Derealization/Depersonalization Spectrum (SCI-DER), the Cambridge Depersonalization Scale (CDS), the Body Sensation Questionnaire (BSQ), the Dissociative Experience Scale (DES), the Panic/Agoraphobic Questionnaire-self report (PAS-SR) for the evaluation of separation anxiety.

**Results:**

The sample was dichotomized into a group with high levels of separation anxiety (3 or more DSM-IV diagnostic items endorsed) *vs*. those with low levels of separation anxiety (less than 3 items endorsed) by PAS-SR ‘Separation Anxiety’ domain scoring. Patients with high separation anxiety scored significantly higher in all DPs scales compared to the low-separation anxiety group. Derealization was significantly correlated with suicidal ideation (p<.001) and overall suicidality (p<.01). Auto-psychic depersonalization, intended as the feeling unfamiliarity of the self in terms of sensation of being an outside observer of one’s mental process, appeared to exert a significant effect on both suicidality (p<.01) and depression (p<.01).

**Conclusions:**

Our findings highlight a link between separation anxiety and DP symptoms. This connection contributes to understanding and evaluating suicidality in individuals with mood and anxiety disorders.

## 1 Introduction

In the fifth edition (revised) of the Diagnostic and Statistical Manual for Mental Disorders (DSM-5-TR) (1), depersonalization refers to the experience of feeling detached from, and as if one is an outside observer of, one’s mental processes, body, or actions. Derealization refers to the experience of feeling detached from one’s surroundings as if one is an outside observer of it. Dissociative symptoms have been documented since 1889, when they were primarily seen in patients then diagnosed with “hysteria” (2, 3). Dissociation is now widely recognized as a transdiagnostic symptom. Dissociation is linked to the onset and maintenance of several disorders, including post-traumatic stress disorder (PTSD), depression, bulimia, and schizophrenia (4, 5) as well as functional somatic syndromes such as fibromyalgia (6). Suicidality, which encompasses a spectrum of thoughts, behaviors, and actions related to suicide, including suicidal ideation, intent, and attempts, has been recognized as a serious risk among individuals with dissociative disorders. While it remains unclear directly increase the risk of suicidality, research suggests that dissociation may contribute to the onset or escalation of suicidal thoughts or behaviors (7, 8). Indeed, research indicates that individuals with depersonalization symptoms are more likely to engage in non-suicidal self-injury (NSSI), experience suicidal ideation, and attempt suicide compared to those with other psychiatric conditions (9–11). Furthermore, individuals with DPs are more likely to engage in NSSI at an earlier age, use a greater variety of self-harm methods, and do so more frequently than those without dissociative symptoms (10). This highlights the significant role dissociation and DPs may play in the development and maintenance of NSSI (12). It is also noteworthy that a high percentage of individuals with DPs report a history of self-harm, with up to 86% engaging in NSSI and as many as 72% attempting suicide at some point in their lives (9, 10, 13–15). While suicidality and self-harm have been studied in various psychiatric conditions, including anxiety disorders, the interplay between dissociation and suicidality remains mainly underexplored. This underscores the need for better recognition of dissociation in clinical practice, as it plays a significant role in the development and maintenance of self-harm and suicidality.

Apart from in Depersonalization Disorder (DPD), depersonalization/derealization symptoms (DPs) have often been considered a non-specific and heterogeneous symptom of anxiety. Some authors have reported that in patients with panic disorder, these symptoms were associated with an earlier age of onset, a history of childhood abuse, a higher prevalence of avoidance, more severe agoraphobia, and higher rates of psychiatric comorbidity (16). Most of these studies were focused on the prevalence of DPs during panic attacks, with ranges from 7.8% to 82.6%, depending on the clinical sample. The heterogeneity of DPs is also evident in other contexts of anxiety.

Other manifestations of DPs consisting of feelings of detachment and a non-specific ‘block’ of all emotions, actions, and perceptions may be connected to separation anxiety, as well. The frantic efforts to avoid abandonment that are typical of individuals with severe separation anxiety may activate a variety of symptoms and behaviors, including the sensation of something being broken within oneself – sometimes described by the patients as a physical sensation – or emotional detachment to worldly concerns (17).

Some researchers endorse the view that, in addition to ineffable feelings of unreality, DPs vary considerably in phenomenology. However, studies published to date in this area have significant limitations: above all, the fact that measures of DPs were limited to assess frequency and endorsement of DSM criteria for DPD. Mula et al. (18, 19) proposed a “spectrum” model of DPs composed of an extensive array of symptoms, including emotional numbing, heightened self-observation, changes in body experience, distortions in the perceptions of time and space, changes in the feeling of agency (with automaton or robot-like experiences, a sensation of lacking control of one’s actions), feelings of having the mind empty of thoughts and memories, an inability to focus and sustain attention, and various types of sensory anesthesia.

Clinically, dissociative symptoms are often challenging to identify, as they are subtle, transient, and less apparent compared to other psychiatric symptoms such as depression or anxiety. This makes it difficult for clinicians to detect dissociative experiences and properly address them in treatment plans.

In addition to the challenges in identifying dissociative symptoms, there is also a gap in understanding how these symptoms interact with specific psychiatric conditions, such as separation anxiety. Individuals with separation anxiety often experience heightened fears of abandonment, which may manifest through emotional detachment and altered perceptions of the self and the world—symptoms that overlap with depersonalization experiences. These symptoms may further increase the risk for suicidality. However, the interplay between dissociation, separation anxiety, and suicidality has yet to be fully explored, making it difficult for clinicians to understand how to treat individuals who exhibit both sets of symptoms effectively.

This gap in knowledge creates challenges for treatment planning, as standard interventions for anxiety and mood disorders may not adequately address the dissociative and suicidal aspects of the patient’s experience. By understanding how depersonalization symptoms interact with separation anxiety and contribute to suicidality, clinicians can develop more comprehensive and targeted interventions.

In the present study, we sought to examine the relationship between depersonalization and derealization (DPs), as assessed through the SCI-DER and other specific scales, separation anxiety, and suicidality in a sample of 156 outpatients with anxiety and mood disorders. Specifically, we aimed to investigate how dissociative symptoms, particularly depersonalization and derealization, relate to suicidality in individuals with and without separation anxiety. As a secondary objective, we aimed to explore the relationship between the frequency and duration of dissociative symptoms and suicidality, to determine whether specific depersonalization domains are more strongly associated with suicidality, and to differentiate between the associations of dissociative symptoms with suicidal ideation versus suicidal behaviors.

While this study is the first to explore the relationship between DP symptoms and separation anxiety, it also addresses several important research gaps that have direct clinical implications. First, while depersonalization and derealization symptoms have been widely studied in trauma-related conditions, their role in mood and anxiety disorders, especially in those with separation anxiety, remains underexplored. Second, understanding the mechanisms by which dissociative symptoms contribute to suicidality—particularly in patients with co-occurring separation anxiety—has important implications for refining clinical interventions. Finally, examining the frequency and duration of dissociative symptoms in relation to suicidality can guide clinicians in assessing the severity and trajectory of symptoms, informing better treatment planning.

By addressing these gaps, the present study aims to enhance our understanding of the complex relationship between dissociative symptoms, separation anxiety, and suicidality, ultimately leading to more effective clinical interventions that can reduce suicidality and improve patient outcomes.

## 2 Methods

### 2.1 Participants

A consecutive sample of adult outpatients presenting for treatment for mood and anxiety disorders at the outpatient clinics of the Department of Psychiatry in Pisa, Italy, from September 2006 to September 2007 were invited to participate in the study. Methodological details have been reported elsewhere (20). Exclusion criteria were severe medical illness, neurological disease, and inability to participate because of severity of psychiatric symptoms. Eligible subjects provided written informed consent after receiving a complete description of the study and having an opportunity to ask questions. The Ethics Committee of the University of Pisa approved all recruitment and assessment procedures.

The study involved a sample of 156 individuals diagnosed with mood and anxiety disorders. All diagnosis were made through the Structured Clinical Interview for DSM-IV Axis (SCID – I). These participants were divided into two groups based on their symptoms of separation anxiety. One group consisted of 100 individuals who met the diagnostic criteria for Separation Anxiety Disorder (high SAD) as outlined in the DSM-IV, which requires at least three symptoms of separation anxiety. The second group included 56 individuals who did not meet this threshold, having fewer than three symptoms of separation anxiety (low-SAD). To assess the presence of such symptoms of separation anxiety, we used the Panic/Agoraphobic Spectrum Questionnaire (PAS), a validated 161-item tool designed to evaluate a wide range of panic and agoraphobic symptoms, including those related to separation anxiety disorder. This questionnaire is comprehensive and allows for the diagnosis of panic disorder, agoraphobia, and separation anxiety disorder (21).

### 2.2 Assessment of suicidality and depression by the Structured Clinical Interview for Mood Spectrum (SCI-MOODS) (Lifetime Version)

Suicidal ideation and behaviors were evaluated by SCI-MOODS (22) items 102 through 107. In particular, the items (102) “you thought that life was not worth living?”; (103) “you hoped that you would not wake up in the morning, or that you would die in an accident or from something like a heart attack or a stroke?”; (104) “you wanted to die or hurt yourself?”; and (105) “if you felt that you wanted to die, did you have a specific plan to hurt or kill yourself?”; were used to assess the presence of suicidal ideation. The items (106) “did you actually try to kill yourself?” and (107) “if you tried to kill yourself, did you require medical attention?” were used to assess suicidal behaviors. The sum of positive answers to these items was labeled a “suicidality score.” To this date, items 102-107 of the SCI-MOODS have previously been used for assessing suicidality in clinical and non-clinical samples (23, 24).

The SCI-MOODS was also employed to assess the dimension of depression through the sum of the items belonging to the *depressive mood* (items 1 to 7), *depressive energy* (items 58 to 66) and *depressive cognition* (items 81 to 107) domains.

### 2.3 Psychometric scales for depersonalization/derealization symptoms

#### 2.3.1 Structured Clinical Interview for Depersonalization/Derealization

The questionnaire includes 49 items exploring the “presence” or “absence” of lifetime spontaneous symptoms of DP organized into four domains: *Derealization* (items 1 – 9), *Somatopsychic depersonalization* (items 10 – 25), *Autopsychic depersonalization* (items 26 – 41), and *Affective depersonalization* (items 42 – 49). The first domain, *Derealization*, refers to an altered experience of the external world and corresponds to the DSM description of derealization. Such an experience is frequently described in terms of visual metaphors (e.g., looking through the fog, a veil between you and the external world). The *Somatopsychic depersonalization* domain describes a variety of changes in body experience, such as lack of body ownership feelings and feelings of disembodiment, which can range from a nonspecific feeling of not being in the body to out-of-body experiences and autoscopic hallucinations, defined as an hallucination characterized by the vision of a double of one’s own body without any changes in bodily self-consciousness (25). Somatosensory distortions, usually affecting the size of body parts, or feeling very light, and lack of body sensations or various types of sensory anesthesia (e.g., hunger, thirst, and pain) are also present. The *Autopsychic depersonalization* domain includes the unfamiliarity of the self in terms of the sensation of being an outside observer of one’s mental process, not being “in charge” of their own behavior or cognitive processes, the automaton-line experience and anomalous subjective recall (e.g., the feeling that personal events happened long ago or had already happened, inability to evoke visual memories of people or places). The *Affective depersonalization* domain explores the patient’s loss of ability to imbue perceptions with emotional feelings but also comprises the loss of affection, pleasure, fear or disgust to situations previously avoided. Items were derived starting from a review of the descriptive psychopathology of DP (18, 26–32). Responses are coded dichotomously (yes/no), and scores are based on the number of positive answers. Symptoms occurring during alcohol or drug use are considered negative. The SCI-DER was initially piloted with patients diagnosed with DPD and refined based on feedback. In its validation study, the scale demonstrated excellent reliability and good concurrent validity with other dissociative experience measures (19).

#### 2.3.2 Body Sensation Questionnaire

The BSQ is a validated self-report psychological assessment tool used to evaluate an individual’s perceptions and experiences related to bodily sensations. It is often used to assess the degree to which individuals experience bodily sensations that may be distressing or related to certain psychological conditions. The instrument was shown to be reliable and fared well on tests of discriminant and construct validity. This instrument was chosen to consider views, expressed in the clinical literature, that DP may be related to anxiety (22) and is frequently reported by patients with agoraphobia (16, 33). The questionnaire was employed to investigate whether subjects whit high or low levels of SAD reported significant differences in the perception of body sensations associated with psychological states.

#### 2.3.3 Dissociative Experiences Scale

The DES is a lifetime 28-item self-rating questionnaire developed explicitly as a screening instrument to identify subjects who are likely to have a dissociative disorder. DES was valid with good internal consistency and excellent test-retest reliability (four weeks = 0.93; eight weeks = 0.90; one year = 0.78). Since its introduction, the DES has been used in hundreds of dissociation studies. Different cut-off scores have been proposed, 20 (34), 25 (35), 30 (36) (for a more detailed review see (37, 38). For the purpose of our study, where we aimed to dimensionally assess dissociative experience, no cut-off scores were needed.

#### 2.3.4 Cambridge Depersonalization Scale

This is composed of 29 items and provides total scores for frequency and duration of depersonalization symptoms over the last 6 months (39). It is a reliable and valid instrument to measure depersonalization symptoms, with strong internal consistency and good reliability (Cronbach alpha = .089) (40). Since the questionnaire was added at a later time, only a partial number of the recruited subjects were evaluated with the CDS.

The selection of assessment tools for this study was driven by several key motivations. For example, the DES was used to screen participants who were likely to exhibit dissociative symptoms, while the SCI-DER expanded the evaluation by addressing a wide range of symptoms associated with dissociation—ranging from typical, fully developed manifestations to more subtle, atypical ones—providing a comprehensive account of the self-reported symptomatology. Additionally, the CSD was included to assess the frequency and duration of dissociative symptoms. The BSQ was utilized to examine the presence of altered perceptions related to bodily sensations, which are often linked to dissociation. Finally, the SCI-MOODS enabled an in-depth investigation of suicidality, encompassing its various components, from suicidal ideation to behavior, and offering an overall measure of suicidality. All psychometric instruments, with the only exception of the CDS, evaluated the presence of life-time psychopathological features. The CDS was the only scale to assess active symptoms at the moment of evaluation. All these scales allowed a comprehensive evaluation of the dissociative symptomatology and of its various dimensions and characteristics that could possibly be associated with suicidal ideation and behaviors.

### 2.4 Statistical analysis

Initially, the sample was divided into two groups based on the presence of at least three symptoms of separation anxiety, as assessed by the PAS-SR. Chi-square analyses were then conducted to compare sociodemographic and diagnostic characteristics between subjects with high and low SAD.

Next, independent t-tests were used to compare SCI-DER, DES, BSQ, CDS and suicidality scores between subjects meeting and not meeting the criteria for SAD.

Two separate Analyses of Variance (ANOVA) were performed. The first analyzed overall suicidality as the dependent variable, with SCI-DER domains as independent variables, to explore which specific derealization domains had a significant effect on suicidality. The second analysis used depression as the dependent variable and the same SCI-DER domains as independent variables to investigate their impact on depressive symptomatology.

Pearson’s correlation coefficients were calculated to examine the relationships between CDS, DES, BSQ and SCI-DER scores and suicidal ideation, behavior, and overall suicidality.

Lastly, we conducted two linear regression analyses. The first analysis used overall suicidality as the dependent variable, with CDS and DER domains as independent variables. The second analysis included overall suicidality as the dependent variable, with CDS, DES, BSQ, and the total SCI-DER score as independent variables.

All statistical analyses were conducted using SPSS version 26.0.

## 3 Results

The sample consisted of 56 subjects exhibiting high levels of separation anxiety symptoms and 100 subjects with low separation anxiety symptoms. The groups did not significantly differ regarding educational level or psychiatric diagnoses (see **Table 1**).

**Table 1:** Sociodemographic and diagnostic comparison between High SAD Group (n=100) and Low SAD Group (n=56) *.

	Low SAD N (%)	High SAD N (%)	Total Sample N (%)	Chi-Square	p
Gender					
**Male**	33(58.9%)	64(64.0%)	97(62.2%)	.393	.531
**Female**	23(41.1%)	36(36.0%)	59(37.8%)		
Level of education					
**Elementary school**	3(5.4%)	12(12.0%)	15(9.6%)	8.409	.078
**Middle school**	19(33.9%)	28 (28%)	47(30.1%)		
**High school**	21(37.5%)	50(50.0%)	71(45.5%)		
**Uncompleted university**	1(1.8%)	0(0.0%)	1(0.6%)		
**Bachelor/master degree**	12(21.4%)	10(10.0%)	22(14.1%)		
Marital status					
**Single**	24(42.8%)	38(38%)	62(39.7%)	5.787	.216
**Married**	26(46.4%)	56(56%)	82(52.6%)		
**Divorced**	2(3.6%)	5(5%)	7(4.9%)		
**Widower/widow**	4(7.2%)	1(1%)	5(3.2%)		
Diagnosis					
**Panic Disorder**	31(56.4%)	60(60%)	91(58.7%)	.194	.660
**No Panic Disorder**	24(43.6%)	40(40%)	64(41.3%)		
**Bipolar Disorder**	42(75.0%)	65(65.0%)	107(68.6%)	1.666	.197
**No Bipolar Disorder**	14(25.0%)	35(35.0%)	49(31.4%)		
**Severe Depression**	41(73.2%)	80(80.0%)	121(77.6%)	1.738	.629
**Moderate Depression**	1(1.8%)	1(1.0%)	2(1.3%)		
**Mild Depression**	2(3.6%)	5(5.0%)	7(4.5%)		
**No Depression**	12(21.4%)	14(14%)	26(16.7%)		
Major Depressive Disorder**	19(34.5%)	35(35.0%)	54(34.8%)	.003	.995
No Major Depressive Disorder**	36(65.5%)	65(65.0%)	101(65.2%)		
Obsessive/Compulsive Disorder**	6(10.9%)	19(19.2%)	25(16.2%)	1.784	.182
No Obsessive/Compulsive Disorder**	49(89.1%)	80(80.8%)	129(83.8%)		
Social anxiety**	0(0.0%)	4(4.0%)	4(2.6%)	2.281	.131
No Social Anxiety**	55(100.0%)	95(96.0%)	150(97.4%)		
PTSD**	1(1.8%)	0(0.0%)	1(0.6%)	1.182	.178
No PTSD**	54(98.2%)	99(100.0%)	153(99.4%)		
Somatization Disorder**	0(0.0%)	2(2%)	2(1.3%)	1.126	.289
No Somatization Disorder**	55(100.0%)	97(98%)	152(98.7%)		

SAD, Separation anxiety disorder; PTSD, Post traumatic stress disorder; *Significant for p<.05; **Evaluation performed on a sub-set of patients.Significant values are reported in bold.

Results from an independent-sample t-test revealed that individuals with separation anxiety scored significantly higher than those without on all SDI-DER domains, with the only exception of the *Affective depersonalization* domain. Additionally, significant differences were found on all CDS domains, as well as the total DES score. On the other hand, no significant differences emerged in the scores obtained at SCI-DER *Affective depersonalization* domain, BSQ total score and in suicidality measures (see **Table 2**).

**Table 2:** SCI-DER, DES, BSQ, CDS and suicidality scores in subjects with and without separation anxiety disorder criteria.

	Total sample N=156		t-test	df	p	Std. Error Difference
	Low SAD (n=56)	High SAD (n=100)				
SCI-DER (n=156)						
**I. Derealization**	2.66 ± 2.29	3.58 ± 2.42	-2.302	153	**.023**	.398
II. Somatopsychic depersonalization**	2.41 ± 2.57	3.56 ± 3.36	-2.375	153	**.019**	.482
**III. Autopsychic depersonalization**	6.20 ± 3.31	7.99 ± 3.57	-3.080	153	**.002**	.582
**IV. Affective depersonalization**	2.63 ± 2.06	3.26 ± 1.96	-1.906	153	.058	.334
DES (n=156)						
**DES total score**	10.01 ± 10.06	17.09 16.02	-3.556	153	**.001**	2.18
BSQ (n=156)						
BSQ total score*	4.75± 2.33	4.09± 1.23	1.943	151	.056	.338
CDS (n=97)						
**Frequency**	9.44 ± 10.23	16.64 ± 15.92	-2.769	100	**.007**	2.60
**Duration**	15.28 ± 17.36	27.38 ± 26.26	-2.272	95	**.008**	4.44
**CDS total score**	24.72 ± 27.27	43.05 ± 41.55	-2.619	95	**.010**	7.00
SCI-MOODS Suicidality						
**Suicidal ideation**	1.46 ± 1.30	1.38 ± 1.27	.373	145	.710	0.22
**Suicidal behaviors**	0.89 ± 1.15	0.89 ± 1.23	.013	98	.990	0.23
**Overall Suicidality**	2.38 ± 2.32	2.30 ± 2.17	.166	98	.868	0.46

Levene’s Test for Equality of Variances: * =.000; **.037; SAD, Separation anxiety disorder; SCI-DER, Structured Clinical Interview for Depersonalization/Derealization; DES, Dissociative Experience Scale; BSQ, Body Sensation Questionnaire; CDS, Cambridge Depersonalization Scale; SCI-MOODS, Structured Clinical Interview for Mood Spectrum.Significant values are reported in bold.

Two separate ANOVA analyses were conducted, with the SCI-DER domains as independent variables. These analyses identified a significant effect of the *Autopsychic Depersonalizatio*n domain on both suicidality and depression, as measured by the SCI-MOODS questionnaire (see **Tables 3**, **4**). However, our analysis failed to report a significant effect of SCI-DER *Affective depersonalization, Somatopsychic depersonalization* and *Derealization* on suicidality. Similarly, no significant effect of SCI-DER *Affective depersonalization* and *Somatopsychic depersonalization* domains on depression emerged.

**Table 3:** ANOVA analyses with suicidality as dependent variable and SCI-DER domains as independent variables carried in the whole sample.

Source	Type III Sum of Squares	df	Mean Square	F	p
**Corrected Model**	370.400	5	74.080	6.923	**.000**
**Intercept**	251.826	1	251.826	23.533	**.000**
**IV. Affective depersonalization**	8.610	1	8.610	.805	.371
**III. Autopsychic depersonalization**	78.596	1	78.596	7.345	**.008**
**II. Somatopsychic depersonalization**	21.508	1	21.508	2.010	.158
**I. Derealization**	2.097	1	2.097	.196	.659
**Separation anxiety PAS criteria**	3.430	1	3.430	.321	572
**Error**	1594.439	149	10.701		
**Total**	7485.000	155			
**Corrected Total**	1964.839	154			

R Squared=0.189; Adjusted R Squared=0.161.

Suicidal ideation and behaviors were evaluated by SCI-MOODS items 102 through 107.Significant values are reported in bold.

**Table 4:** ANOVA analyses with depression as dependent variables and SCI-DER domains as independent variables carried in the whole sample.

Source	Type III Sum of Squares	df	Mean Square	F	p
**Corrected Model**	1930.060	5	386.012	12.724	**.000**
**Intercept**	113.678	1	113.678	3.747	.055
**Affective depersonalization**	38.905	1	38.905	1.282	.259
**Autopsychic depersonalization**	369.568	1	369.568	12.182	**.001**
**Somatopsychic depersonalization**	34.350	1	34.350	1.132	.289
**Derealization**	121.823	1	121.823	4.015	**.047**
Separation anxiety* PAS-SR criteria	55.799	1	55.799	1.839	.177
**Error**	4520.417	149	30.338		
**Total**	19168.000	155			
**Corrected Total**	6450.477	154			

SCI-DER, Structured Clinical Interview for Derealization/Depersonalization Spectrum; PAS-SR, panic agoraphobic spectrum – self report; Separation anxiety* PAS- SR criteria: separation anxiety symptomatology measured with PAS-SR first domain; R Squared = =.299; Adjusted R Squared = 0.276.Significant values are reported in bold.

Pearson’s correlation analysis revealed a strong, statistically significant positive relationship between the CDS *Duration* and *Frequency* subscales, as well as the total CDS score, and both suicidal ideation and overall suicidality. However, no significant correlations were observed between CDS scores and suicidal behaviors. Similarly, DES total score and all SCI-DER domain and total scores were significantly and positively correlated with all suicidality measures, with the only exception of SCI-DER *Derealization* domain that did not correlate significantly with suicidal behaviors (see **Table 5**).

**Table 5:** Pearson’s correlation between CDS, BSQ, DES and SCI-DER scores and suicidality measured via SCI-MOODS.

	Suicidal Ideation	Suicidal Behaviors	Global Suicidality
CDS			
**Frequency**	.341**	.148	.214*
**Duration**	.324**	.121	.263**
**CDS total score**	.324**	.126	.258*
BSQ			
**BSQ total score**	.114	-.010	.035
DES			
**DES total score**	.369**	.360**	.403**
SCI-DER			
**I. Derealization**	.300**	.184	.266**
**II.Somatopsychic depersonalization**	.302**	.315*	.353**
**III.Autopsychic depersonalization**	.325**	.266*	.344**
**III. Affective depersonalization**	.310**	.204*	.301**
**SCI-DER total score**	.366**	.298**	.383**

CDS, Cambridge Depersonalization Scale; BSQ, Body Sensation Questionnaire; SCI-DER, Structured Clinical Interview for Depersonalization/Derealization; DES, Dissociative Experience Scale; SCI-MOODS: *Structured Clinical Interview for Mood Spectrum*; *p<.05; **p<.01.Significant values are reported in bold.

The first linear regression performed with suicidality as dependent variable and SCI-DER and CDS domains score as independent variable showed a predictive value of SCI-DER *Autopsychic depersonalization* domain and CDS *Duration* domain on overall suicidality (see **Table 6**).

**Table 6:** Linear regression analysis performed with suicidality as dependent variable and SCI-DER and CDS domains score as independent variable.

	B(S.E.)	Beta	t	p
*constant*	.546 (.230)		2.378	.019*
**Autopsychic depersonalization**	.118(.028)	.325	4.120	<.001*
R^2^:.105; Adjusted R^2^:.099				
*constant*	1.119(.177)		6.307	<.001*
**Duration**	.017(.005)	.324	3.209	.002*
R^2^:.105; Adjusted R^2^:.095				

*Significant for p<.05.Significant values are reported in bold.

Results from the linear regression performed with suicidality as dependent variable and SCI-DER, CDS, BSQ and DES total scores as independent variable showed a predictive value of SCI-DER total score on overall suicidality (see **Table 7**).

**Table 7:** Linear regression analysis performed with suicidality as dependent variable and SCI-DER, CDS, BSQ and DES total scores as independent variable.

	B (S.E.)	Beta	t	p
*constant*	.557 (.252)		2.206	.030*
**SCI-DER total score**	.052 (.012)	.412	4.196	<.001*

*R^2^:.170; Adjusted R^2^:.160;* SCI-DER, Structured Clinical Interview for Depersonalization/Derealization.Significant values are reported in bold.

* Signifant for p<.05.

## 4 Discussion

In this study, we aimed to establish the relationship between DP symptoms and separation anxiety in a sample of outpatients with anxiety and mood disorders, and to examine the potential clinical impact of such symptoms in terms of suicidality. Our initial hypothesis that patients with high scores on separation anxiety would present more frequent and severe DP symptoms than those with low levels of separation anxiety was confirmed by the results of the study.

Interestingly, significant differences between the group with separation anxiety versus the group without were found on both the SCI-DER and the CDS scales used to assess DP symptoms. The concordance in findings between the two scales highlights an essential link between separation anxiety and elevated depersonalization/derealization experiences, suggesting that, among individuals with affective and anxiety disorders, those with separation anxiety may be more prone to experience disruptions in their sense of self or understanding of reality than those without. In these individuals, intense emotional distress related to fear of abandonment or separation may overwhelm the individual’s ability to process emotions, leading to DP as a defense mechanism or a side effect. Similarly, these individuals’ propensity to disengage from reality, indicated by increased “derealization” domain symptoms, maybe a response to the intense anxiety triggered by separation. From this perspective, our data are consistent with the view that dissociation may serve as a maladaptive strategy in patients with detachment experiences to escape overwhelming emotional states (41–43). Alternatively, these symptoms may be linked through core dysregulated attachment experiences (44). However, it is important to note that not all DP domains were significantly related to separation anxiety. For instance, the SCI-DER *Affective Depersonalization* domain did not show significant differences between the groups, suggesting that while depersonalization may be present, it does not uniformly manifest across all domains in individuals with separation anxiety. Similarly, no significant differences were observed in the BSQ total score or suicidality measures between the groups, highlighting the complexity of the relationship between separation anxiety and these outcomes. These findings suggest that while DP may be an important symptom in some individuals with separation anxiety, it does not universally correlate with all aspects of the disorder, nor does it consistently contribute to suicidality in every case.

Another interesting finding is the phenomenological richness and duration of DP symptoms, as evaluated by the CDS, related to the separation anxiety group compared to those in other groups. This reflects the construct of the CDS designed to assess the multifaceted phenomenology of depersonalization and the duration of single symptoms (39). In the CDS validation article, the authors argued that in patients with anxiety disorders or depression, symptoms of depersonalization are secondary to the primary condition and usually are fleeting and last seconds or a few minutes. However, in other individuals, depersonalization symptoms (e.g., anhedonia or experiences of mind emptiness) tended to linger for hours. In our study, the group with separation anxiety resembles the latter group of individuals described by Sierra & Berrios (39). Nonetheless, our results are limited by lack of a PTSD measure. This is consistent with routine clinical experience with patients with separation anxiety who, not rarely, report enduring cognitive and emotional distortions, such as feelings of unreality and affective depersonalization, with, in some cases, critical clinical consequences when their relationships with significant persons are experienced as endangered (23). Despite these interesting findings, we did not observe significant effects of certain SCI-DER domains, such as *Somatopsychic Depersonalization* and *Derealization*, on suicidality or depression. This indicates that while DP symptoms, particularly in the context of separation anxiety, may contribute to emotional distress, not all dimensions of dissociation are equally linked to suicidality or depressive symptomatology. This aligns with the idea that dissociation is a multifaceted phenomenon, and its relationship with suicidality and depression may vary depending on the specific characteristics of the dissociative experience (42, 43).

Our findings revealed significant correlations of both CDS subscale and total scores with suicidal ideation and overall suicidality, as well as a positive correlation between DES total score and all SCI-DER domain and total scores with all suicidality measures, with the only exception of SCI-DER *Derealization* domain that did not correlate significantly with suicidal behaviors. These results suggest that active dissociative symptoms were correlated with a higher risk for suicide. Even though individuals with dissociative disorders frequently report a history of suicide attempts (45), few studies have systematically compared the suicidality of patients with DP symptoms to those without. Despite the scant data available, a growing body of research suggests that depersonalization may significantly contribute to suicidality, in line with our findings. For instance, a study by Golubović et al. (46) utilized the CDS to examine derealization in a sample of depressed patients. Their results indicated that individuals with derealization symptoms were eight times more likely to experience active suicidal ideation and eleven times more likely to report passive suicidal thoughts compared to those without derealization symptoms. Similarly, Foote et al. (9) compared self-harming behaviors and suicidality in patients with and without dissociative symptoms. Their results reported not only that the presence of a dissociative disorder was strongly associated with all measures of self-harm and suicidality but also that the presence of a dissociative diagnosis was significantly predictive of a history of multiple suicide attempts. Although dissociation is often overlooked in studies of suicidality, these results highlight the need to thoroughly investigate dissociative symptoms, such as depersonalization and derealization, regardless of primary diagnosis.

The connection between DPs, and in particular autopsychic DP and the duration of the symptomatology, and increased suicide risk, as reflected by the regression analyses, is in line with the available literature. Indeed, meta-analyses of dissociative disorders reveal that individuals with chronic DP or derealization exhibit significantly higher rates of suicidal ideation, particularly when these symptoms coexist with depression or anxiety (42, 43). Individuals experiencing this form of DP often feel profoundly disconnected from their sense of self, leading to diminished personal agency and a loss of meaning. This estrangement may weaken innate protective mechanisms, such as self-preservation, making individuals more vulnerable to suicidal ideation. Moreover, research highlights that DP frequently overlaps with emotional numbness, hopelessness, and ruminative thinking, which are recognized precursors to suicidal thoughts due to their interference with healthy emotional processing and regulation, leaving individuals trapped in a state of unrelieved psychological distress. Dissociation also is likely to make these attachment-dysregulated people feel more estranged and unable to trust others, heightening a sense of aloneness (47). The aforementioned emotional blunting can mimic or exacerbate anhedonia, intensifying feelings of emptiness and despair (48). Our data suggest these processes may be more pronounced among individuals with separation anxiety, as an additional component to their primary diagnosis, than in those without. It is noteworthy that in a previous study, we found that separation anxiety acted as an important mediating factor in the relationship between depression and suicidality (23).

This pattern of persistent symptoms resembles the clinical picture seen in patients with borderline personality disorder (BPD), a condition often characterized by chronic dissociation and recurrent suicidality (1). In BPD, dissociative symptoms frequently emerge as part of broader affective instability, with individuals experiencing episodes of depersonalization or derealization, particularly during periods of emotional distress or interpersonal conflict (49, 50). Like those with separation anxiety, individuals with BPD may engage in dissociative coping strategies, particularly when dealing with overwhelming emotions or fears of abandonment. The parallel between separation anxiety and BPD is significant because both conditions are associated with emotional dysregulation and attachment disorders, which may increase vulnerability to dissociation and suicidality (51). Furthermore, both groups tend to report similar dissociative symptoms, such as feelings of unreality, emotional numbness, and identity confusion, which may exacerbate interpersonal difficulties and intensify suicidal ideation.

Although we did not specifically measure childhood adversity in our study, it is important to consider how this factor could have influenced the results. The presence of separation anxiety in individuals who may have experienced early attachment disruptions or trauma could enhance vulnerability to dissociative symptoms, making them more prone to feeling disconnected from themselves or reality during times of stress (12). This would suggest that the relationship between separation anxiety and DP symptoms and both NSSI and suicidality might be further exacerbated by a history of childhood adversity, as the individual’s ability to manage emotional distress could be compromised by their previous experiences of trauma (12).

Several limitations must be acknowledged. The most glaring is the omission of any measure of PTSD. PTSD has been shown to arise more commonly among people with separation anxiety, and conversely, separation anxiety can arise after PTSD (52). It may well be that associations between derealization/depersonalization and separation anxiety that were observed in this study arose from this link. First, the study was not originally designed with a focus on suicide. Therefore, there are no specific scales to evaluate risk or other aspects of the phenomenology of suicidality. However, the assessment of suicidality by the SCI-MOODS was utilized in numerous previous studies, and experience with this instrument has resulted in reliable and reflecting clinical reality (24, 53). A second limitation is that, in the present study the intensity of single current separation anxiety symptoms as for instance possible in some degree in the Adult Separation Anxiety Questionnaire (ASA-27) (54, 55) could not be assessed. For all items evaluating separation anxiety, the presence of the symptom has been scored as “absent or “present”. The presently observed association between separation anxiety, DP symptoms, and suicidality cannot be generalized to patients with psychiatric diagnoses other than affective disorders or the general population. Third, the present analyses could not be corrected for the potential influence of early trauma. This is of relevance in light of earlier studies demonstrating adverse childhood experiences, such as maltreatment, to constitute a risk factor for either suicidality, dissociative experiences, or separation anxiety (56). Thus, there might be a mediating effect of one of these conditions on the link between childhood trauma and suicidality. Furthermore, the CDS was completed only by a sub-set of participants, possibly impacting the validity of the comparison.

In conclusion, separation anxiety is an important clinical dimension, often with roots in childhood, but likely to manifest across the lifespan. This study indicates that when patients with mood or anxiety disorders are categorized based on the severity of concomitant separation anxiety, there are significant clinical and phenomenological differences. In our study, individuals with high scores on separation anxiety had more frequent and severe depersonalization/derealization symptoms than those with low scores on separation anxiety. The presence of DP symptoms, in turn, was predictive of a higher risk of suicidality. Clinicians should screen for separation anxiety disorder and other secondary symptoms, such as DPs, in adult patients with mood and anxiety disorders. This may have important clinical implications for treatment interventions and for reducing suicide risk effectively.

## Data Availability

The original contributions presented in the study are included in the article/supplementary material, further inquiries can be directed to the corresponding author/s.
